# Modified Stress Score and Sympathetic–Parasympathetic Ratio Using Ultra-Short-Term HRV in Athletes: A Novel Approach to Autonomic Monitoring

**DOI:** 10.3390/jfmk10030310

**Published:** 2025-08-12

**Authors:** Andrew D. Fields, Matthew A. Mohammadnabi, Michael V. Fedewa, Michael R. Esco

**Affiliations:** Department of Kinesiology, The University of Alabama, 620 Judy Bonner Drive, Tuscaloosa, AL 35401, USA; adfields1@crimson.ua.edu (A.D.F.); amohammadnabi@crimson.ua.edu (M.A.M.); mvfedewa@ua.edu (M.V.F.)

**Keywords:** autonomic cardiac measurement, athlete monitoring, heart rate variability, training recovery, sympathetic nervous system, measurement methodology

## Abstract

**Background:** Monitoring autonomic balance provides valuable insights into recovery status and physiological readiness, both of which are essential for performance optimization in athletes. The Stress Score (SS) and Sympathetic–Parasympathetic Ratio (SPS), derived from Poincaré plot heart rate variability (HRV) indices, have been proposed as practical markers of sympathetic activity and overall autonomic balance. However, these traditional calculations often require lengthy recordings and specialized software, limiting their feasibility in field settings. This study introduces modified versions of these metrics derived from ultra-short-term (1 min) time–domain HRV recordings: the Modified Stress Score (MSS) and Modified Sympathetic–Parasympathetic Ratio (MSPS). **Methods:** Competitive male athletes (*n* = 20, age = 21.2 ± 2.1 year, height = 183.6 ± 8.9 cm, weight = 79.2 ± 10.3 kg) completed a maximal exercise test with HRV recorded before and after exercise. **Results:** Following natural log-transformation, MSS and MSPS demonstrated strong correlations with SS and SPS across all time points (*r* = 0.87–0.94, all *p* < 0.01) and displayed the expected physiological responses to exercise and recovery. **Conclusions:** These findings suggest that MSS and MSPS are practical, accessible tools for assessing autonomic balance in athletes. Their application may enhance our ability to monitor recovery status, guide individualized training strategies, and optimize performance in applied sport settings.

## 1. Introduction

Over the past two decades, heart rate variability (HRV) has become an important measure of stress, recovery, and readiness to perform in the realm of physical training. Currently, a common feature among wearable technology, the root mean square of successive normal-to-normal interval differences (RMSSD), has arguably become the primary metric for monitoring long-term adaptation and recovery status [1,2]. While RMSSD serves as a valuable objective indicator of internal load, its physiological underpinnings primarily reflect the parasympathetic branch of the autonomic nervous system [3]. Thus, sympathetic activity may not be directly captured by relying solely on RMSSD. Although parasympathetic and sympathetic activities often exhibit an inverse relationship, this is not always the case, as both branches can be co-activated and suppressed under specific conditions [4]. Consequently, the interpretation of RMSSD should be complemented by additional metrics to provide a more comprehensive understanding of autonomic balance in the context of athlete recovery and performance [5,6].

Poincaré plot is a nonlinear HRV method that has been used to evaluate the interplay between sympathetic and parasympathetic activity. This method plots each RR interval (peak R-wave of one QRS complex on an electrocardiogram to the next) against the subsequent interval, creating an elliptical-shaped scatterplot [7]. The standard deviation calculated from the perpendicular distance of the points from the line of identity (SD1) indicates short-term variability and parasympathetic activity, while the standard deviation of the points along the length of the line of identity (SD2) reflects overall variability [8]. Although SD2 and the SD1:SD2 ratio have been controversially proposed as markers of sympathetic activity and the autonomic balance of parasympathetic-to-sympathetic control, respectively [9], recent reports challenge these claims, noting that both SD1 and SD2 tend to increase or decrease in parallel [7,10].

To account for these shortcomings, Naranjo-Orellana et al. [10] introduced two metrics that are calculated from the Poincaré plot indices: the Stress Score (SS) and the Sympathetic–Parasympathetic ratio (SPS). The SS, calculated as 1/SD2, was proposed as a marker of sympathetic activity, based on its inverse association with parasympathetically mediated HRV indices [10]. Additionally, the SPS is derived from the relationship between SS and SD1, and is viewed as a more accurate indicator of autonomic balance compared to the traditional SD1:SD2 ratio [10]. Since their inception, SS and SPS have been validated for their intended purposes in several subsequent studies [5,11,12,13].

Despite their potential, specialized software that many may require Poincaré plot indices to be derived can be impractical in field settings due to time constraints and logistical challenges. In contrast, time–domain metrics such as RMSSD and SDNN (the standard deviation of normal-to-normal RR intervals) are more accessible and strongly correlate with SD1 and SD2, respectively [14,15], making them viable proxies for calculating SS and SPS. Cicconne et al. mathematically showed that RMSSD and SD1 are identical metrics that are simply attained in different ways (one using time–domain methods and one using nonlinear methods) [16], while Hoshi et al. demonstrated an R^2^ of 0.86, accounting for the variance between SDNN and SD2 [14].

These relationships are not only statistical, but also physiological. RMSSD and SD1 both reflect short-term, high-frequency variability driven primarily by parasympathetic vagal modulation, which explains their mathematical equivalence and interchangeable use in the literature [3,14,16]. Likewise, SDNN and SD2 capture global variability across the entire recording, including contributions from both sympathetic and parasympathetic input, which justifies their use as surrogates for overall autonomic modulation [3,14,16]. Therefore, substituting RMSSD for SD1 and SDNN for SD2 provides a physiologically and statistically coherent foundation for constructing modified versions of SS and SPS, particularly in contexts where time–domain metrics are more practical to obtain than nonlinear indices [3,14,16].

In an effort to reconcile some of the impracticalities of Poincaré plot-based HRV metrics in field settings, a recent study by Salazar-Martinez et al. [17] demonstrated that SS could be calculated from SDNN in a group of athletes during exercise. However, a modified SPS calculation using RMSSD as a substitute for SD1 was not explored. The study also relied on SDNN values derived from 5 min heart rate recordings, which are often impractical in field applications. Therefore, a logical next step is to examine whether both SS and SPS can be calculated directly from 1 min ultra-short recordings using time–domain metrics alone. This simplified approach would eliminate the need for regression modeling and nonlinear analysis, potentially increasing the practicality of autonomic balance monitoring in applied sport settings. Consequently, numerous studies have validated the use of ultra-short 1 min recordings for RMSSD and SDNN [18,19,20]. It remains unknown, however, whether modified calculations of both SS and SPS from these ultra-short time–domain metrics are feasible, particularly in athletic populations.

An acute bout of maximal exercise offers an excellent model for studying autonomic balance due to the known shift toward sympathetic dominance with the return of parasympathetic control during recovery [21]. This creates an ideal context to evaluate the utility of SS and SPS indices derived from both traditional and modified approaches. Most HRV metrics tend to decrease during exercise and increase following exercise, which is reflective of the parasympathetic nervous response [22,23]. Indeed, if modified versions of both SS and SPS are respective indicators of sympathetic activity and autonomic balance, then both markers should increase during maximal exercise and decrease during the recovery stages. Therefore, the purpose of this study was twofold: (1) develop modified versions of the stress score (MSS) and sympathetic–parasympathetic ratio (MSPS) by using ultra-short-term (1 min) recordings of RMSSD and SDNN and determine their correlation with traditional measures (SS and SPS, respectively); (2) evaluate the effects of an acute maximal exercise bout on both the modified (MSS and MSPS) and traditional (SS and SPS) measures in competitive athletes. For comparative purposes, correlations and trends across the bout of maximal exercise were also analyzed for the component HRV metrics (i.e., SD1, SD2, RMSSD, and SDNN).

## 2. Materials and Methods

### 2.1. Study Design

**Figure 1:**
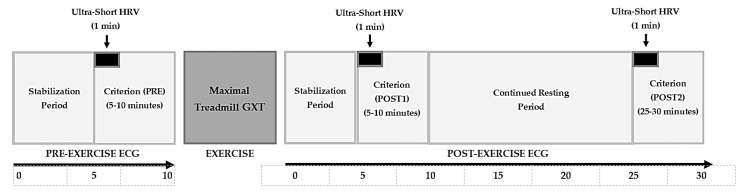
ECG recording timeline schematic for pre- and post-exercise recordings, as well as criterion and ultra-short analyzed HRV segments highlighted at PRE, POST1, and POST2.

The two hypotheses of the study were as follows: (1) significant correlations will be revealed between SS and MSS and between SPS and MSPS; and (2) the modified and traditional measures will display similar temporal trends in response to an acute bout of maximal exercise. To test these hypotheses, 20 male athletes completed a maximal graded exercise test on a treadmill. For 10 min before and 30 min after the exercise, the participants assumed the supine position while electrocardiographic (ECG) recordings were obtained for HRV analysis. SD1 and SD2 were recorded from 5 min ECG epochs and were used to calculate SS and SPS at the following time points: between minutes 5–10 of pre-exercise (PRE), between minutes 5–10 post-exercise (POST1), and between minutes 25–30 post-exercise (POST2). MSS and MSPS were calculated from ultra-short-term SDNN and RMSSD derived from 1 min ECG segments at the following time points: 5–6 min of PRE, 5–6 min of POST1, and 25–26 min of POST2 (Figure 1). Correlations were determined between the traditional and modified values at each time point, as well as between their trends from pre- to post-exercise.

### 2.2. Participants

Data from a convenience sample of twenty male (age = 21.2 ± 2.1 year, height = 183.6 ± 8.9 cm, weight = 79.2 ± 10.3 kg) soccer and basketball athletes from the National Association for Intercollegiate Athletics were retroactively analyzed for this study. Data collection occurred as close to waking from sleep as possible for each subject (between 7:00 am and 11:00 am). Participants reported to the lab following an overnight fast, and avoided the consumption stimulants (e.g., caffeine) and depressants (e.g., alcohol) for 12 h prior to the visit. They also avoided strenuous exercise over the previous 24 h. This study was approved by the University Institutional Review Board for Human Participants.

### 2.3. Procedures

Each participant completed a maximal graded exercise test using the standard Bruce Treadmill protocol (Trackmaster, Full Vision, Inc., Carrollton, TX, USA). This involved increasing speed and grade every 3 min until volitional fatigue, with the initial stage beginning at 1.7 mph at 10% grade. When the subjects reached a point of maximal exhaustion, the treadmill speed and grade were decreased to 1.5 mph and 1.5% grade, respectively, for a 2 min active cool-down period.

Before the bout of exercise, each subject assumed a supine position on an athletic training table in a dimly lit, quiet laboratory with temperature and humidity at approximately 22 °C and 50%, respectively. While in this position, heart rate and rhythm were assessed utilizing ECG, with three surface electrodes placed in a modified Lead II arrangement following proper site preparation (e.g., cleaned with alcohol swabs). The electrodes were attached by wire leads to a Biopac MP100 data acquisition system (Goletta, CA, USA). Once the ECG began, participants remained motionless in this position for 10 min. Following active cool-down from the GXT, subjects were allowed a 2 min timeframe to return to the athletic training table. The subjects once again assumed a supine position and remained motionless for 30 min total.

### 2.4. HRV Acquisition and Modified Calculation

The recorded ECGs were divided into three segments: 5–10 min pre-exercise (PRE); 5–10 min post-exercise (POST1); and 25–30 min post-exercise (POST2). Each segment was visually inspected, and if less than three ectopic beats were identified, they were removed and replaced by subsequent normal cycles. However, if three or more were found in any of the segments, the subject was excluded from analysis (*n* = 0).

All R-R interval data were processed using Kubios HRV Scientific 4.0.0 (Kubios Oy, Kuopio, Finland). Artifact and noise correction was performed using the medium automatic threshold setting, which applies a threshold-based beat-correction algorithm to identify and replace artifacts with interpolated values. This approach is consistent with recommended preprocessing standards for time–domain HRV metrics at rest, as outlined in the methodological guidelines by Catai et al. [24]. R-R intervals were obtained from ECG recordings sampled at 250 Hz.

The Poincaré plot indices of SD1, SD2, and SD1:SD2 were derived from each of the three 5 min ECG segments. These nonlinear metrics formed the basis for the traditional indices, where SD2 was used to calculate the Stress Score (SS) and SD1 was used to calculate the Sympathetic–Parasympathetic ratio (SPS) following the original procedures developed by Naranjo-Orellana et al. [10], as shown in Equations (1) and (2):

(1) SS = 1000 × (1/SD2)

(2) SPS = SS/SD1

In contrast, the modified indices were derived from ultra-short (1 min) time–domain HRV recordings. Specifically, SDNN was used as a surrogate for SD2 and RMSSD as a surrogate for SD1, based on their established mathematical and physiological correspondence. The Modified Stress Score (MSS) and Modified Sympathetic–Parasympathetic ratio (MSPS) were therefore calculated using the same equations, but with time–domain inputs, as shown in Equations (3) and (4):

(3) MSS = 1000 × (1/SDNN)

(4) MSPS = MSS/RMSSD

### 2.5. Statistical Analysis

A post hoc power analysis was conducted using G*Power 3.1 (Heinrich Heine University Düsseldorf, Düsseldorf, Germany). For a repeated-measures analysis of variance (RMANOVA) with 3 time points (α = 0.017, η^2^ = 0.25, correlation among repeated measures = 0.7), the achieved power (1-β) was 0.87, suggesting that the sample size of 20 participants was sufficient to detect moderate-to-large effect sizes. Data were statistically analyzed with SPSS Statistics version 29.0 (Chicago, IL, USA). The Shapiro–Wilk test indicated that the assumption of normality was violated for all the studied HRV parameters (*p*  <  0.05). Accordingly, natural log-transformations (ln) were applied to the HRV variables to normalize the data before statistical analysis, which is a common procedure in HRV measurement [1]. Importantly, the specific HRV metrics (SD1, SD2, RMSSD, and SDN) were first log-transformed and are, therefore, reported and analyzed as lnSD1, lnSD2, lnRMSSD, and lnSDNN. The traditional and modified stress scores (SS and MSS) and Sympathetic–Parasympathetic ratios (SPS and MSPS) were initially calculated using the raw HRV values and then log-transformed, resulting in lnSS, lnSPS, lnMSS, and lnMSPS. To maintain clarity, log-transformed values (denoted with “ln”) are used consistently when presenting statistical findings. However, when discussing the physiological meaning or broader implications of these metrics in the Discussion, the original metric names (e.g., RMSSD, SS, SPS, etc.) are used to preserve conceptual clarity.

Pearson correlation coefficients (r) were used to determine the relationships between lnSS and lnMSS and between lnSPS and lnMSPS at PRE, POST1, and POST2, as well as their relationships with each selected HRV metric. RMANOVA procedures and subsequent paired t-tests were used to determine the potential differences from the pre- to post-exercise time points for lnSS, lnMSS, lnSPS, and lnMSPS, as well as for each raw HRV metric, using a Bonferroni correction (*p*  <  0.017) for each paired comparison. In addition, Cohen’s d effect sizes were calculated for each pairwise comparison. Hopkins scale for determining the magnitude of the effect size was used, where 0.0–0.2 = “*trivial*”, 0.2–0.6 = “*small*”, 0.6–1.2 = “*moderate*”, 1.2–2.0 = “*large*”, >2.0 = “*very large*” [25]. Intraclass correlation coefficients (ICC) for repeated measures with 95% confidence intervals (CIs) were used to assess the degree to which lnSS and lnMSS, as well as lnSPS and lnMSPS, followed similar trends across PRE, POST1, and POST2 time points. This method allows for determining the association between two variables that have different numerical values over time. The thresholds used to qualitatively interpret the r and ICC values were based on Hopkins [25] using the following criteria: *r*  <  0.00–0.10 was *trivial*; 0.11–0.30 was *small*; 0.31–0.50 was *moderate*; 0.51–0.70 was *large*; 0.71–0.90 was *very large*; and >0.91 was *nearly perfect*. Statistical significance for the correlation procedures was determined as *p*  <  0.05.

Please note that although the equations for SS and MSS, as well as for SPS and MSPS, were mathematically similar, they were derived from different HRV variables. Specifically, SS and SPS were calculated using SD1 and SD2 from the Poincaré plot, while MSS and MSPS were adapted using SDNN and RMSSD. This fundamental distinction results in the modified scores being on a different numerical scale than their traditional counterparts. Understanding this difference is important when interpreting the statistical analyses used to compare the traditional and modified values.

## 3. Results

**Figure 2:**
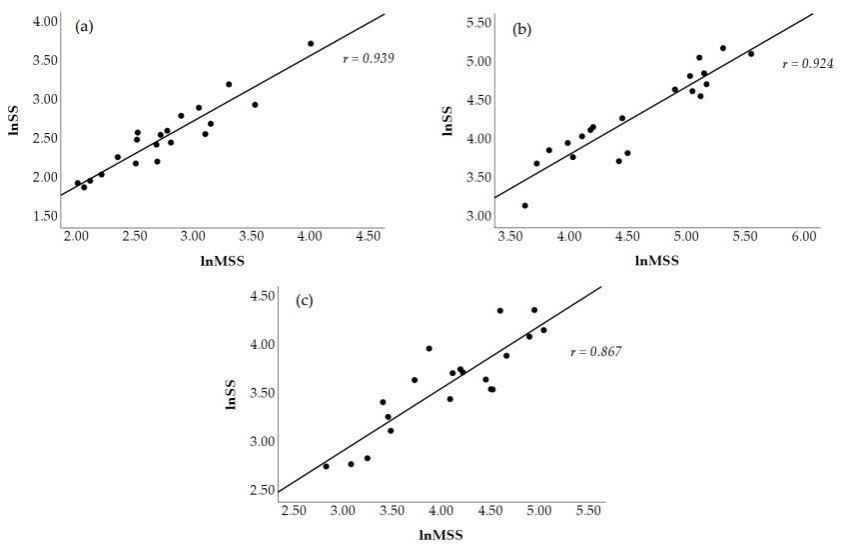
Scatterplot representation of the Pearson product movement correlations between traditional (lnSS) and modified (lnMSS) natural log-transformed stress scores at PRE (**a**), POST1 (**b**), and POST2 (**c**).

**Figure 3:**
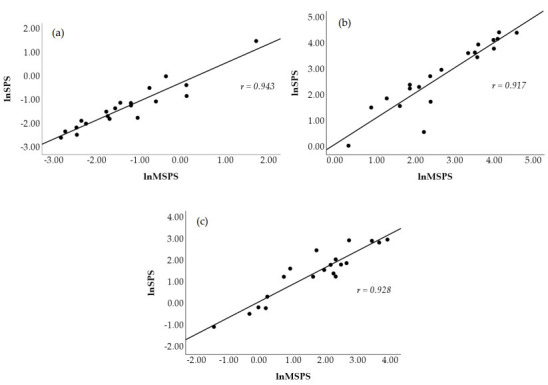
Scatterplot representation of the Pearson product movement correlation between traditional (lnSPS) and modified (lnMSPS) natural log-transformed sympathetic–parasympathetic ratio at PRE (**a**), POST1 (**b**), and POST2 (**c**).

Figure 2 visually displays a scatterplot of the correlations between natural log-transformed SS and MSS at all time points. The correlations coefficients between these two metrics were *r* = 0.939 (*p* < 0.001, nearly perfect, Figure 2a) at PRE, *r* = 0.924 (*p* < 0.001, nearly perfect, Figure 2b) at POST1, and *r* = 0.867 (*p* < 0.001, very large, Figure 2c) at POST2. Figure 3 shows the scatterplot of the correlations between natural log-transformed MSPS and SPS. These correlation coefficients were *r* = 0.943 (*p* < 0.001, nearly perfect, Figure 3a) at PRE, *r* = 0.917 (*p* < 0.001, nearly perfect, Figure 3b) at POST1, and *r* = 0.928 (*p* < 0.001, nearly perfect, Figure 3c) at POST2.

**Figure 4:**
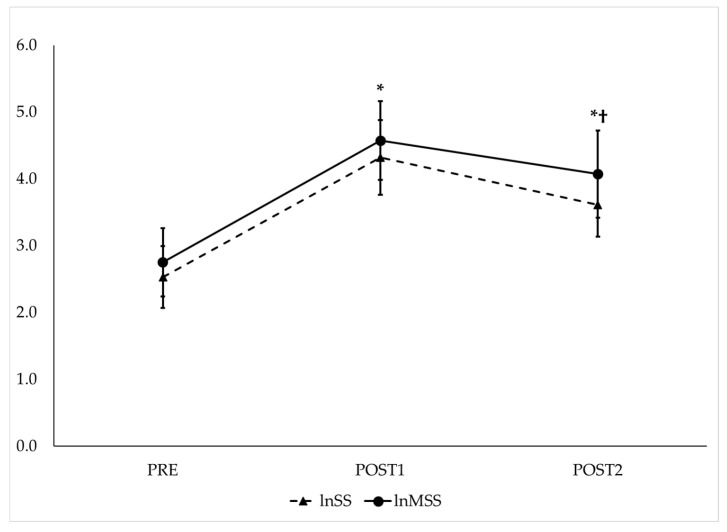
Observed trends of traditional (lnSS—dashed line) and modified natural log-transformed stress scores (lnMSS—solid line) before and after maximal GXT. PRE = HRV recording pre-GXT from 5 to 10 min, POST1 = HRV recording post-GXT from 5 to 10 min, POST2 = HRV recording post-GXT from 25 to 30 min. * Significantly different from PRE (*p* < 0.017). † Significantly different from POST1 (*p* < 0.017).

**Figure 5:**
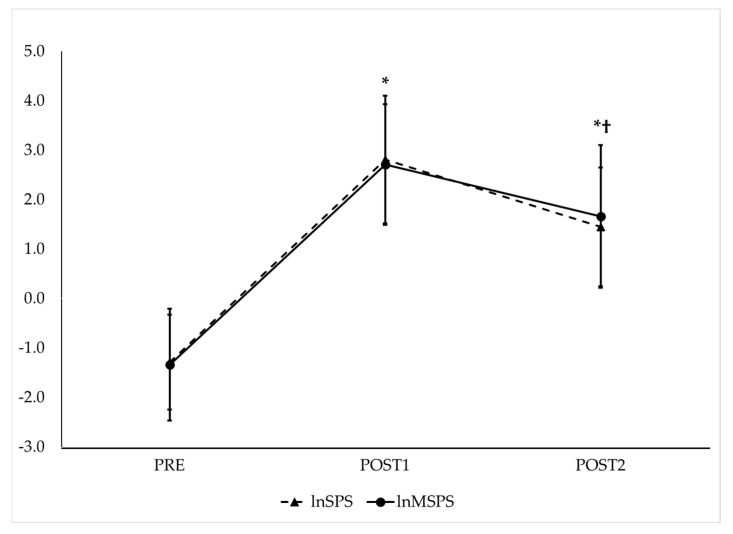
Observed trends of traditional (lnSPS—dashed line) and modified natural log-transformed sympathetic–parasympathetic ratio (lnMSPS—solid line) before and after maximal GXT. PRE = HRV recording pre-GXT from 5 to 10 min, POST1 = HRV recording post-GXT from 5 to 10 min, POST2 = HRV recording post-GXT from 25 to 30 min. * Significantly different from PRE (*p* < 0.017). † Significantly different from POST1 (*p* < 0.017). Note: SPS and MSPS values were derived from log-transformed components. This transformation can yield negative values when the parasympathetic component exceeds the sympathetic component, as is often the case under resting conditions.

RMANOVA with follow-up pairwise comparisons showed that lnSS and lnMSS (Figure 4), as well as lnSPS and lnMSPS (Figure 5), increased from PRE to POST1 and then decreased from POST1 to POST2 while remaining significantly higher than PRE (*p* < 0.017 for all and *moderate* to *very large* effect sizes, as presented in Table 1). The repeated measures ICC procedures revealed *very large* correlations between the trends in lnSS and lnMSS (ICC = 0.82; 95% CI = 0.66–0.92, *p* < 0.001) and between the trends in lnSPS and lnMSPS (ICC = 0.84; 95% CI = 0.70–0.93, *p* < 0.001) across the three time points.

In addition, each HRV metric (lnSD1, lnSD2, lnSD1:SD2, lnRMSSD, and lnSDNN), decreased from PRE to POST1 and increased from POST1 to POST2, while remaining lower when compared to PRE (*p* < 0.017 for all and *moderate* to *very large* effect sizes, as presented in Table 1), with the exception of lnSD1:SD2 remaining suppressed at POST2. The correlation coefficients between each HRV parameter and the traditional and modified values are shown in Table 2. The majority of the correlations are inverse and are qualified as large to nearly perfect. At all times points, lnSD1, lnSD2, lnRMSSD, and lnSDNN displayed larger inverse correlations with lnMSS and lnMSPS than they did with lnSS and lnSPS.

## 4. Discussion

The present study provides novel insights into the use of ultra-short-term time–domain HRV metrics for modified calculations of the Poincaré plot-derived SS and SPS in athletes. Please note that the specific findings of this study discussed below reflect log-transformed values (e.g., lnRMSSD, lnSS, lnSPS, etc.), whereas the original metric names (e.g., RMSSD, SS, SPS, etc.) are used when referring to general physiological concepts or broader interpretations. The findings show that, using 1 min recordings of lnSDNN and lnRMSSD as proxies for the criterion 5 min indices of lnSD1 and lnSD2, there are significant and very large-to-nearly perfect correlation coefficients between lnSS and lnMSS and between lnSPS and lnMSPS at the PRE, POST1, and POST2 time points. Furthermore, the modified and traditional measures display similar trends across the three time points, which are supported by the very large and significant repeated measures ICC values. The collective findings indicate that modified calculations were just as effective as the traditional values at capturing the shifts in autonomic control in response to maximal exercise. This suggests that MSS and MSPS could be valuable for monitoring sympathetic activity and overall autonomic balance in athletes, which may complement the utility of the parasympathetically mediated RMSSD without creating added methodological inconveniences. Because these metrics can be derived from brief recordings using common time–domain outputs, MSS and MSPS may be particularly well-suited for integration into applied monitoring strategies in team sport environments, wearable devices, or recovery tracking platforms. Their ease of calculation from 1 min recordings offers a feasible solution for practitioners seeking rapid assessments of autonomic status.

The SS and SPS were introduced by Naranjo-Orellana et al. [10] as tools to better assess autonomic balance through HRV analysis, particularly since SD1 and SD2 increase or decrease in a concurrent fashion, refuting previous claims that SD1:SD2 ratio is a marker of parasympathetic-to-sympathetic balance [5,26]. The present results support the findings from Naranjo-Orellana et al. [10]. In response to the acute bout of maximal exercise, lnSD1, lnSD2, and lnSD1:SD2, as well as lnRMSSD and lnSDNN, all decreased from PRE to POST1. Although lnSD1:SD2 remained depressed, the other HRV metrics slightly increased at POST2 from POST1, while remaining lower than their PRE values. This pattern appears to more accurately reflect decreasing parasympathetic activity in response to maximal exercise and its rebound during recovery [18,27]. Thus, sympathetic responses do not appear to be captured from examining these individual HRV metrics alone.

However, in response to the acute bout of maximal exercise, lnSS and lnSPS increased from PRE to POST1, followed by decreases from POST1 to POST2 while remaining higher than PRE levels. These responses are consistent with the expected effects of physical exertion on autonomic balance, marked by a shift from parasympathetic to sympathetic dominance [28,29]. For instance, decreases in RMSSD and SDNN with subsequent increases in SS and SPS have been demonstrated immediately following vertical kilometer trail running [15]. In addition, Miranda-Mendoza et al. [29] demonstrated congruent decreases in RMSSD and increases in SS following six competitive matches in high-level handball players, likely reflecting the shift from parasympathetic-to-sympathetic dominance that occurs with accumulated fatigue. Therefore, the collective findings support the notion that SS and SPS are reliable indicators of sympathetic activity and parasympathetic-to-sympathetic balance, respectively [10]. Furthermore, they reinforce the value of incorporating SS and SPS alongside traditional HRV metrics, such as RMSSD, to enhance the understanding of autonomic regulation and recovery processes in athletes.

Unfortunately, the calculation of SS and SPS necessitates Poincaré plotting and longer timeframes for recording that may not be convenient within sportive settings. However, both RMSSD and SDNN have been shown to provide valid measurements when recorded over a 1 min duration [18,20,27]. In addition, previous findings demonstrated that RMSSD and SDNN are strongly correlated with SD1 and SD2, respectively [14,15]. Therefore, the current study calculated MSS and MSPS from the ultra-short recordings of the two time–domain indices. The findings show that lnMSS and lnMSPS followed the same respective trends as lnSS and lnSPS in response to the maximal graded exercise test by increasing from PRE to POST1 while returning toward, but still elevated from, PRE levels at POST2, which was verified by the significant and *very large* repeated-measures ICCs. Furthermore, most of the HRV metrics (i.e., lnRMSSD, lnSDNN, lnSD1 and lnSD2) displayed significant inverse correlations with lnMSS and lnMSPS at each time point (i.e., PRE, POST1 and POST2). Interestingly, each HRV parameter displayed larger correlation coefficients with lnMSS and lnMSPS (very large-to-nearly perfect) than they did with lnSS and lnSPS (large-to-nearly perfect) at all time points. The only exception was that lnSD1:SD2 at PRE and POST1 was not significantly correlated with lnMSS and lnMSPS, likely due to the unclear association between this ratio and autonomic activity [10].

These results align with recent reports from Salazar-Martínez et al. [17], who also estimated SS from SDNN values. However, several key differences highlight how the current study extends their work. First, Salazar-Martínez et al. [17] calculated SDNN values from 5 min HRV recordings, whereas our study focused on ultra-short 1 min recordings. Second, their study did not explore SPS estimation. Third, their estimation of SS involved a two-step process: (1) predicting SD2 from SDNN using a regression equation, and (2) estimating SS from the predicted SD2 values. In contrast, our study aimed to simplify this process by directly substituting SD2 with ultra-short SDNN and SD1 with ultra-short RMSSD. Although the results provide different numerical values than the traditional methods, the modified approaches allowed for the direct calculation of MSS and MSPS from 1 min time–domain recordings, eliminating the need for an intermediate regression model. Despite this simplification, we observed strong correlations and similar PRE-to-POST2 trends between the traditional metrics (lnSS and lnSPS) and the modified indices (lnMSS and lnMSPS). By demonstrating that MSS and MSPS can be directly derived from ultra-short time–domain metrics, our study provides a more practical and accessible method for assessing autonomic function in athletes. Therefore, the modified scores (MSS and MSPS) appear to be as effective as traditional metrics (SS and SPS) in indicating sympathetic activity and autonomic balance in response to exercise. However, because of their simplified acquisition directly from ultra-short recordings and streamlined calculations, MSS and MSPS offer a practical advantage, particularly for monitoring daily recovery status and readiness to perform in athletes [30,31,32].

Although the findings of this study are promising, several limitations should be acknowledged. First, the sample consisted exclusively of young male collegiate athletes, which enhanced internal control, but limits the generalizability of the results. Given the known sex-based and age-related differences in autonomic function and HRV patterns, it is important that future studies include female participants, as well as athletes across a broader age range, training status, and levels of competition. This would help determine whether the modified metrics (MSS and MSPS) behave consistently across more diverse populations. Additionally, the current study focused on post-exercise recovery following a single acute bout of maximal exercise. Thus, further research is needed to explore the utility of MSS and MSPS for monitoring longitudinal training adaptations, including periods of overload, tapering, and potential maladaptation. While controlled laboratory conditions were used to enhance standardization and internal validity, this approach may not reflect the variability encountered in field-based applications. Furthermore, while the purpose of the study was to examine acute within-day autonomic responses, we acknowledge that ultra-short HRV metrics can vary across days. Future studies should, therefore, investigate the validity, sensitivity, and reliability of MSS and MSPS in real-world sport environments, such as during training cycles, competitive seasons, or recovery monitoring in applied athletic settings.

Nonetheless, the present results highlight the potential utility of MSS and MSPS as practical and reliable indicators of sympathetic activity and autonomic balance in athletes. The findings demonstrate that these modified scores, derived from ultra-short-term recordings of RMSSD and SDNN, exhibit strong correlations with their Poincaré plot-derived counterparts, while displaying similar responses to acute maximal exercise. While further research is needed to investigate the long-term effects of training interventions, this study provides a strong foundation for using both MSS and MSPS, alongside parasympathetically mediated HRV markers, when monitoring recovery and adaptation in athletes.

## 5. Conclusions

The findings of this study support the growing body of research advocating for the adoption of ultra-short time–domain HRV measures to improve the feasibility of monitoring autonomic responses in athletes. The present results suggest that MSS and MSPS may provide a more robust assessment of autonomic balance when collected alongside other HRV metrics, such as RMSSD. For instance, elevated MSS and MSPS values may indicate heightened sympathetic overdrive and insufficient recovery, while lower values may signal readiness for higher training intensities or competition. The simplified acquisition methods of MSS and MSPS make them particularly suitable for use among practitioners in field-based applications, such as daily recovery assessments and monitoring readiness in athletes. Ultimately, this approach aligns with the increasing emphasis on precision monitoring to tailor training programs to individual athletes’ physiological responses, enhancing both performance and recovery outcomes.

## Figures and Tables

**Table 1:** Changes in natural log-transformed HRV metrics from PRE, POST1, and POST2 surrounding treadmill GXT.

				Effect Sizes for Pairwise Comparisons (Cohen’s d)		
	PRE	POST1	POST2	PRE v POST1	PRE v POST2	POST1 v POST2
Nonlinear						
lnSD1	3.82 ± 0.54	1.51 ± 0.76 ^*^	2.16 ± 0.74 *†	3.51 *Very Large*	2.56 *Very Large*	−0.87 *Moderate*
lnSD2	4.38 ± 0.46	2.59 ± 0.56 ^*^	3.30 ± 0.48 *†	3.49 *Very Large*	2.30 *Very Large*	−1.36 *Large*
lnSD1:SD2	0.59 ± 0.18	0.37 ± 0.21 ^*^	0.34 ± 0.13 *	1.12 *Moderate*	1.59 *Large*	0.17 *Trivial*
lnSS	2.53 ± 0.46	4.32 ± 0.56 ^*^	3.61 ± 0.48 *†	−3.49 *Very Large*	−2.30 *Very Large*	1.36 *Very Large*
lnSPS	−1.29 ± 0.96	2.81 ± 1.29 ^*^	1.45 ± 1.20 *†	−3.61 *Very Large*	−2.52 *Very Large*	1.09 *Moderate*
Time–Domain						
lnRMSSD	4.09 ± 0.66	1.87 ± 0.65 ^*^	2.41 ± 0.81 *†	3.39 *Very Large*	2.27 *Very Large*	−0.74 *Moderate*
lnSDNN	4.16 ± 0.51	2.34 ± 0.59 ^*^	2.84 ± 0.65 *†	3.30 *Very Large*	2.26 *Very Large*	−0.81 *Moderate*
lnMSS	2.75 ± 0.51	4.57 ± 0.59 ^*^	4.07 ± 0.65 *†	−3.30 *Very Large*	−2.26 *Very Large*	0.81 *Moderate*
lnMSPS	−1.34 ± 1.13	2.71 ±1.22 ^*^	1.66 ± 1.44 *†	−3.44 *Very Large*	−2.32 *Very Large*	0.79 *Moderate*

* Significantly different from PRE (*p* < 0.017). † Significantly different from POST1 (*p* < 0.017). Values are listed as means ± standard deviation. Note: SPS and MSPS values were derived from log-transformed components. This transformation can yield negative values when the parasympathetic component exceeds the sympathetic component, as is often the case under resting conditions.

**Table 2:** Correlation coefficients of the relationships between each of the natural log-transformed HRV metrics and the stress scores and parasympathetic–sympathetic ratios before (PRE), 5–10 min after (POST1), and 25–30 min (POST2) after a graded exercise test.

	lnSS			lnMSS			lnSPS			lnMSPS		
	*r*	*p*	*Qualifier*	*r*	*p*	*Qualifier*	*r*	*p*	*Qualifier*	*r*	*p*	*Qualifier*
PRE												
lnSD1	−0.72	<0.001	*Very large*	−0.92	<0.001	*Nearly Perfect*	−0.60	0.005	*Large*	−0.95	<0.001	*Nearly Perfect*
lnSD2	−0.83	<0.001	*Very large*	−0.94	<0.001	*Nearly Perfect*	−0.59	0.007	*Large*	−0.86	<0.001	*Very Large*
lnSD1:SD2	−0.14	0.565	*Small*	−0.30	0.202	*Small*	−0.30	0.200	*Small*	−0.34	0.137	*Small*
lnRMSSD	−0.63	<0.001	*Large*	−0.89	<0.001	*Very Large*	−0.53	0.017	*Large*	−0.98	<0.001	*Nearly Perfect*
lnSDNN	−0.76	0.003	*Very large*	−1.00	<0.001	*Nearly Perfect*	−0.55	0.013	*Large*	−0.96	<0.001	*Nearly Perfect*
POST1												
lnSD1	−0.61	0.004	*Large*	−0.77	<0.001	*Very Large*	−0.60	0.005	*Large*	−0.85	<0.001	*Very Large*
lnSD2	−0.85	<0.001	*Very large*	−0.92	<0.001	*Nearly Perfect*	−0.79	<0.001	*Very Large*	−0.94	<0.001	*Nearly Perfect*
lnSD1:SD2	−0.21	0.372	*Small*	−0.15	0.526	*Small*	−0.29	0.221	*Small*	−0.24	0.311	*Small*
lnRMSSD	−0.70	<0.001	*Large*	−0.91	<0.001	*Nearly Perfect*	−0.67	0.001	*Large*	−0.98	<0.001	*Nearly Perfect*
lnSDNN	−0.84	<0.001	*Very large*	−1.00	<0.001	*Nearly Perfect*	−0.80	<0.001	*Very Large*	−0.98	<0.001	*Nearly Perfect*
POST2												
lnSD1	−0.72	<0.001	*Very large*	−0.85	<0.001	*Very Large*	−0.64	0.002	*Large*	−0.92	<0.001	*Nearly Perfect*
lnSD2	−0.88	<0.001	*Very large*	−0.88	<0.001	*Very Large*	−0.77	<0.001	*Very Large*	−0.91	<0.001	*Nearly Perfect*
lnSD1:SD2	−0.51	0.021	*Large*	−0.51	0.021	*Large*	−0.53	0.017	*Large*	−0.46	0.041	*Large*
lnRMSSD	−0.67	0.001	*Large*	−0.94	<0.001	*Nearly Perfect*	−0.59	0.007	*Large*	−0.99	<0.001	*Nearly Perfect*
lnSDNN	−0.77	<0.001	*Very large*	−1.00	<0.001	*Nearly Perfect*	−0.67	0.001	*Large*	−0.98	<0.001	*Nearly Perfect*

## Data Availability

The raw data supporting the conclusions of this article will be made available by the authors on request.

## Abbreviations

The following abbreviations are used in this manuscript:

HR	Heart Rate
HRV	Heart Rate Variability
RR Interval	The time between the peak R-wave of one QRS complex on an ECG to the next
SS	Stress Score
SPS	Sympathetic–Parasympathetic Ratio
MSS	Modified Stress Score
MSPS	Modified Sympathetic–Parasympathetic Ratio
RMSSD	Root Mean Squared of the Successive Differences of R-R Intervals
SDNN	Mean of Standard Deviations of all Normal-to-Normal R-R Intervals
SD1	Poincaré plot standard deviation perpendicular to the line of identity
SD2	Poincaré plot standard deviation along the line of identity
ECG	Electrocardiography
GXT	Graded Exercise Test(s)
PRE	5 min pre-GXT ECG recording (minutes 5–10 of a 10 min recording
POST1	5 min post-GXT ECG recording (minutes 5–10 of a 30 min recording)
POST2	5 min post-GXT ECG recording (minutes 25–30 of a 30 min recording)
RMANOVA	Repeated-Measures Analysis of Variance
ICC	Intraclass Correlation Coefficient
CI	Confidence Interval

## References

[1] Buchheit M. (2014). Monitoring training status with HR measures: Do all roads lead to Rome?. Front. Physiol..

[2] Plews D.J., Laursen P.B., Stanley J., Kilding A.E., Buchheit M. (2013). Training Adaptation and Heart Rate Variability in Elite Endurance Athletes: Opening the Door to Effective Monitoring. Sports Med..

[3] TFESCNASP Electrophysiology (1996). Heart Rate Variability: Standards of Measurement, Physiological Interpretation, and Clinical Use. Circulation.

[4] Berntson G.G., Bigger J.T., Eckberg D.L., Grossman P., Kaufmann P.G., Malik M., Nagaraja H.N., Porges S.W., Saul J.P., Stone P.H. (1997). Heart rate variability: Origins, methods, and interpretive caveats. Psychophysiology.

[5] Navarro-Lomas G., De-la O.A., Jurado-Fasoli L., Castillo M.J., Femia P., Amaro-Gahete F.J. (2020). Assessment of autonomous nerve system through non-linear heart rate variability outcomes in sedentary healthy adults. PeerJ.

[6] Shaffer F., McCraty R., Zerr C.L. (2014). A healthy heart is not a metronome: An integrative review of the heart’s anatomy and heart rate variability. Front. Physiol..

[7] Karmakar C.K., Khandoker A.H., Voss A., Palaniswami M. (2011). Sensitivity of temporal heart rate variability in Poincaré plot to changes in parasympathetic nervous system activity. Biomed. Eng. Online.

[8] Brennan M., Palaniswami M., Kamen P. (2001). Do existing measures of Poincaré plot geometry reflect nonlinear features of heart rate variability?. IEEE Trans. Biomed. Eng..

[9] Tulppo M.P., Mäkikallio T.H., Takala T.E., Seppänen T., Huikuri H.V. (1996). Quantitative beat-to-beat analysis of heart rate dynamics during exercise. Am. J. Physiol..

[10] Orellana J.N., Torres B.d.l.C., Cachadiña E.S., de Hoyo M., Cobo S.D. (2015). Two New Indexes for the Assessment of Autonomic Balance in Elite Soccer Players. Int. J. Sports Physiol. Perform..

[11] de la Cruz Torres B., Albornoz Cabello M., García Bermejo P., Naranjo Orellana J. (2016). Autonomic responses to ultrasound-guided percutaneous needle electrolysis of the patellar tendon in healthy male footballers. Acupunct. Med..

[12] García Bermejo P., de la Cruz Torres B., Naranjo Orellana J., Albornoz Cabello M. (2017). Autonomic activity in women during percutaneous needle electrolysis. Eur. J. Integr. Med..

[13] Proietti R., di Fronso S., Pereira L.A., Bortoli L., Robazza C., Nakamura F.Y., Bertollo M. (2017). Heart Rate Variability Discriminates Competitive Levels in Professional Soccer Players. J. Strength Cond. Res..

[14] Hoshi R.A., Pastre C.M., Vanderlei L.C.M., Godoy M.F. (2013). Poincaré plot indexes of heart rate variability: Relationships with other nonlinear variables. Auton. Neurosci..

[15] Muñoz-Pérez I., Varela-Sanz A., Lago-Fuentes C., Navarro-Patón R., Mecías-Calvo M. (2022). Central and Peripheral Fatigue in Recreational Trail Runners: A Pilot Study. Int. J. Environ. Res. Public Health.

[16] Ciccone A.B., Siedlik J.A., Wecht J.M., Deckert J.A., Nguyen N.D., Weir J.P. (2017). Reminder: RMSSD and SD1 are identical heart rate variability metrics. Muscle Nerve.

[17] Salazar-Martínez E., Naranjo-Orellana J., Sarabia-Cachadiña E. (2024). Heart rate variability: Obtaining the stress score from SDNN values. Isokinet. Exerc. Sci..

[18] Esco M.R., Flatt A.A., Williford H.N. (2017). Postexercise heart rate variability following treadmill and cycle exercise: A comparison study. Clin. Physiol. Funct. Imaging.

[19] Shaffer F., Meehan Z.M., Zerr C.L. (2020). A Critical Review of Ultra-Short-Term Heart Rate Variability Norms Research. Front. Neurosci..

[20] Esco M.R., Flatt A.A. (2014). Ultra-short-term heart rate variability indexes at rest and post-exercise in athletes: Evaluating the agreement with accepted recommendations. J. Sports Sci. Med..

[21] Stanley J., Peake J.M., Buchheit M. (2013). Cardiac Parasympathetic Reactivation Following Exercise: Implications for Training Prescription. Sports Med..

[22] Cristina Barreto A., Medeiros A.P., da Silva Araujo G., Vale R., Vianna J.M., Alkimin R., Serra R., Leitão L., Reis V.M., da Silva Novaes J. (2023). Heart rate variability and blood pressure during and after three CrossFit^®^ sessions. Retos.

[23] Pober D.M., Braun B., Freedson P.S. (2004). Effects of a Single Bout of Exercise on Resting Heart Rate Variability. Med. Sci. Sports Exerc..

[24] Catai A.M., Pastre C.M., Godoy M.F.D., Silva E.D., Takahashi A.C.D.M., Vanderlei L.C.M. (2020). Heart rate variability: Are you using it properly? Standardisation checklist of procedures. Braz. J. Phys. Ther..

[25] Hopkins W.G. (2002). A New View of Statistics: A Scale of Magnitudes for Effect Statistics. https://www.sportsci.org/resource/stats/effectmag.html.

[26] Rahman S., Habel M., Contrada R.J. (2018). Poincaré plot indices as measures of sympathetic cardiac regulation: Responses to psychological stress and associations with pre-ejection period. Int. J. Psychophysiol..

[27] Esco M.R., Williford H.N., Flatt A.A., Freeborn T.J., Nakamura F.Y. (2018). Ultra-shortened time-domain HRV parameters at rest and following exercise in athletes: An alternative to frequency computation of sympathovagal balance. Eur. J. Appl. Physiol..

[28] Abellán-Aynés O., Manonelles P., Alacid F. (2021). Cardiac Parasympathetic Withdrawal and Sympathetic Activity: Effect of Heat Exposure on Heart Rate Variability. Int. J. Environ. Res. Public Health.

[29] Miranda-Mendoza J., Reynoso-Sánchez L.F., Hoyos-Flores J.R., Quezada-Chacón J.T., Naranjo J., Rangel-Colmenero B., Hernández-Cruz G. (2020). Stress score and lnRMSSD as internal load parameters during competition. Rev. Int. Med. Cienc. Act. Fis. Deporte.

[30] Bechke E., Kliszczewicz B., McLester C., Tillman M., Esco M., Lopez R. (2020). An examination of single day vs. multi-day heart rate variability and its relationship to heart rate recovery following maximal aerobic exercise in females. Sci. Rep..

[31] Medeiros A.R., Leicht A.S., Michael S., Boullosa D. (2021). Weekly vagal modulations and their associations with physical fitness and physical activity. Eur. J. Sport. Sci..

[32] Williams T.D., Esco M.R., Fedewa M.V., Bishop P.A. (2020). Inter- and Intra-Day Comparisons of Smartphone-Derived Heart Rate Variability across Resistance Training Overload and Taper Microcycles. Int. J. Environ. Res. Public Health.

